# Butyrate Enhances Disease Resistance of Chickens by Inducing Antimicrobial Host Defense Peptide Gene Expression

**DOI:** 10.1371/journal.pone.0027225

**Published:** 2011-11-04

**Authors:** Lakshmi T. Sunkara, Mallika Achanta, Nicole B. Schreiber, Yugendar R. Bommineni, Gan Dai, Weiyu Jiang, Susan Lamont, Hyun S. Lillehoj, Ali Beker, Robert G. Teeter, Guolong Zhang

**Affiliations:** 1 Department of Animal Science, Oklahoma State University, Stillwater, Oklahoma, United States of America; 2 Department of Animal Science, Iowa State University, Ames, Iowa, United States of America; 3 Animal Parasitic Diseases Laboratory, Animal and Natural Resources Institute, United States Department of Agriculture-Agricultural Research Service, Beltsville, Maryland, United States of America; Auburn University, United States of America

## Abstract

Host defense peptides (HDPs) constitute a large group of natural broad-spectrum antimicrobials and an important first line of immunity in virtually all forms of life. Specific augmentation of synthesis of endogenous HDPs may represent a promising antibiotic-alternative approach to disease control. In this study, we tested the hypothesis that exogenous administration of butyrate, a major type of short-chain fatty acids derived from bacterial fermentation of undigested dietary fiber, is capable of inducing HDPs and enhancing disease resistance in chickens. We have found that butyrate is a potent inducer of several, but not all, chicken HDPs in HD11 macrophages as well as in primary monocytes, bone marrow cells, and jejuna and cecal explants. In addition, butyrate treatment enhanced the antibacterial activity of chicken monocytes against *Salmonella enteritidis*, with a minimum impact on inflammatory cytokine production, phagocytosis, and oxidative burst capacities of the cells. Furthermore, feed supplementation with 0.1% butyrate led to a significant increase in HDP gene expression in the intestinal tract of chickens. More importantly, such a feeding strategy resulted in a nearly 10-fold reduction in the bacterial titer in the cecum following experimental infections with *S. enteritidis*. Collectively, the results indicated that butyrate-induced synthesis of endogenous HDPs is a phylogenetically conserved mechanism of innate host defense shared by mammals and aves, and that dietary supplementation of butyrate has potential for further development as a convenient antibiotic-alternative strategy to enhance host innate immunity and disease resistance.

## Introduction

Host defense peptides (HDPs), also known as antimicrobial peptides, are present in virtually all species of life and constitute a critical component of the innate immunity [1], [2], [3], [4], [5]. Defensins and cathelicidins represent two major families of HDPs in vertebrates [6], [7], [8], [9], [10], [11]. While defensins are categorized by the presence of six conserved cysteine residues in the C-terminal mature sequence [6], [7], [8], [11], all cathelicidins consist of a conserved cathelin domain in the pro-sequence with a highly diversified C-terminal mature sequence [9], [10]. The chicken genome was recently found to encode a total of 14 β-defensins known as AvBD1-14 [12], [13], [14] and four cathelicidins, namely fowlicidins 1–3 [12], [15], [16] and cathelicidin-B1 [17]. All AvBDs are densely clustered on chicken chromosome 3q [13], [14], whereas cathelicidin genes are located on chromosome 2p [16], [17]. Both chicken AvBDs and cathelicidins are expressed in a wide range of tissues, with cathelicidins expressed most abundantly in the bone marrow or bursa [15], [16], [17] and β-defensins in the liver and throughout the digestive, respiratory, and reproductive tracts [12], [14].

HDPs possess broad-spectrum antimicrobial activities against bacteria, protozoa, enveloped virus, and fungi mainly through direct binding and lysis of microbial membranes [5], [18]. Because of such physical interactions, it is extremely difficult for pathogens to develop resistance to HDPs. Many chicken HDPs such as AvBD9 (formally known as gallinacin-6) and cathelicidin B1 have been found to possess potent antibacterial activities against a broad range of bacteria including *Salmonella* [16], [19], [20], [21], [22], [23], [24], [25]. Besides direct microbicidal activities, HDPs have a strong capacity to modulate the innate immune response by inducing chemotaxis and activation of various types of leukocytes [2], [4]. Because of these pleiotropic effects, HDPs have been actively explored as a new class of therapeutic agents against antibiotic-resistant microbes and other inflammatory diseases [2], [5].

Butyrate, a major species of short-chain fatty acids produced by bacterial fermentation of undigested carbohydrates in the intestine [26], [27], was recently found to be capable of inducing HDP expression in humans and rabbits [28], [29], [30]. To test whether butyrate can augment HDP gene expression in a non-mammalian species, we studied the effect of butyrate on HDP gene expression and the antibacterial activity of monocytes in the chicken. Furthermore, we examined the effect of supplementing butyrate in the feed on the titer of *Salmonella enteritidis* in the cecum following experimental infections. We concluded that butyrate-mediated induction of HDP synthesis is phylogenetically conserved in both mammals and aves. Additionally, butyrate may be further exploited as a cost-effective feed or food additive in enhancing host immunity and disease resistance.

## Materials and Methods

### Ethics statement

This study was carried out in strict accordance with the recommendations in the Guide for the Care and Use of Laboratory Animals of the National Institutes of Health. All animal procedures reported herein were approved by the Institutional Animal Care and Use Committee of Oklahoma State University under protocol no. AG0610. Prior to sample collection, chickens were euthanized by an intramuscular injection of a cocktail of ketamine/xylazine, followed by cervical dislocation to minimize pain.

### Isolation, culture, and stimulation of chicken cells and intestinal tissue explants

Chicken HD11 macrophage cells [31] were cultured in complete RPMI 1640 containing 10% fetal bovine serum (FBS), 100 U/ml penicillin, and 100 µg/ml streptomycin, and seeded at 2×10^6^ cells/well in 6-well cell culture plates overnight, prior to stimulation with different concentrations of sodium butyrate (Sigma) in duplicate and incubated at 37°C and 5% CO_2_ for indicated times. Chicken peripheral blood mononuclear cells (PBMCs) were isolated from EDTA-anticoagulated venous blood of adult layers through gradient centrifugation using Histopaque 1077 (Sigma). Monocytes were obtained by seeding PBMCs at 3×10^7^ cells/well in 6-well plates overnight and washing off non-adherent cells twice with calcium- and magnesium-free Hank's balanced salt solution (HBSS). Monocytes were replenished with fresh complete RPMI 1640 prior to stimulation with sodium butyrate. Bone marrow cells were collected from femur bones of 1- to 2-week-old broiler chickens, lysed of erythrocytes, and cultured at 1×10^7^ cells in 60-mm tissue culture dishes in RPMI 1640 containing 20 mM HEPES, 10% FBS, 100 U/ml penicillin, and 100 µg/ml streptomycin, followed by butyrate stimulation. Jejunal and cecal explants were obtained by washing thoroughly a segment of the jejunum and cecum of 1- to 2-week-old broiler chickens with cold HBSS containing 50 µg/ml of gentamicin, followed by slicing in a series of 0.5-cm long segments and placing individually in 6-well tissue culture plates in RPMI 1640 containing 20 mM HEPES, 10% FBS, 100 U/ml penicillin, 100 µg/ml streptomycin, and 50 µg/ml gentamicin. Jejunal and cecal explants were cultured at 37°C and 5% CO_2_ in the presence of different concentrations of sodium butyrate in duplicate for 24 h.

### Real-time RT-PCR analysis of chicken HDP gene expression

Following treatment with sodium butyrate, chicken cells and tissue explants were lysed in Tri Reagent (Sigma) for extraction of total RNA. The first-strand cDNA was synthesized from 300 ng of total RNA using QuantiTect Reverse Transcription Kit (Qiagen) in a total volume of 4 µl. Real-time PCR was then performed using QuantiTect SYBR Green PCR kit (Qiagen) and MyiQ Real-Time PCR Detection System (Bio-Rad) in 10 µl reactions containing 1/40 or 1/20 of the first-strand cDNA and gene-specific primers for 14 AvBDs, 4 chicken cathelicidins, and multiple cytokines (Table 1) as described [16], [25], [32]. PCR cycling conditions were 95°C for 10 min, followed by 45 cycles of 94°C for 15 sec, 55°C for 20 sec, and 72°C for 30 sec. The specificity of PCR reaction was confirmed by the melt curve analysis. The gene expression levels were quantified using the comparative ΔΔCt method with the glyceraldehyde-3-phosphate dehydrogenase (*GAPDH*) gene as a reference for normalization.

**Table 1:** Primer sequences of chicken host defense peptides and cytokines for real time PCR^a^.

Gene	Forward primer	Reverse primer	Product size(bp)	
			cDNA	Gene
AvBD1	ATGCGGATCGTGTACCTGCTC	CTGCTTGGGATGTCTGGCTCT	219	1197
AvBD2	CTCTCTCCTCTTCCTGGCAC	GAGGGGTCTTCTTGCTGCTG	265	1122
AvBD3	ATGCGGATCGTGTACCTGCTC	CAGAATTCAGGGCATCAACCTC	196	2379
AvBD4	CATCTCAGTGTCGTTTCTCTGC	ACAATGGTTCCCCAAATCCAAC	321	899
AvBD5	CTGCCAGCAAGAAAGGAACCTG	TGAACGTGAAGGGACATCAGAG	300	1100
AvBD6	AGGATTTCACATCCCAGCCGTG	CAGGAGAAGCCAGTGAGTCATC	249	1203
AvBD7	CTGCTGTCTGTCCTCTTTGTGG	CATTTGGTAGATGCAGGAAGGA	230	665
AvBD8	TTCTCCTCACTGTGCTCCAA	AAGGCTCTGGTATGGAGGTG	124	383
AvBD9	GCAAAGGCTATTCCACAGCAG	AGCATTTCAGCTTCCCACCAC	211	1802
AvBD10	TGGGGCACGCAGTCCACAAC	ATCAGCTCCTCAAGGCAGTG	298	2285
AvBD11	ACTGCATCCGTTCCAAAGTCTG	TCGGGCAGCTTCTCTACAAC	301	1299
AvBD12	CCCAGCAGGACCAAAGCAATG	GTGAATCCACAGCCAATGAGAG	335	731
AvBD13	CATCGTTGTCATTCTCCTCCTC	ACTTGCAGCGTGTGGGAGTTG	175	4514
AvBD14	CTCCTGTTTCTTGTTCTCCTG	CACTTTGCCAGTCCATTGTAG	149	501
Cath-B1	CCGTGTCCATAGAGCAGCAG	AGTGCTGGTGACGTTCAGATG	170	251
Fowlicidin-1	GCTGTGGACTCCTACAACCAAC	GGAGTCCACGCAGGTGACATC	261	882
Fowlicidin-2	CAAGGAGAATGGGGTCATCAG	CGTGGCCCCATTTATTCATTCA	221	584
Fowlicidin-3	GCTGTGGACTCCTACAACCAAC	TGGCTTTGTAGAGGTTGATGC	352	1095
IL-1β	GACATCTTCGACATCAACCAG	CCGCTCATCACACACGACAT	215	384
IL-8	GCTGATCGTAAAGGCACTTATG	GTGAAAGGTGGAAGATGGAATG	159	727
IL-12p40	GACCCACCTCAATGTCAGTATG	GCCCAGTCTTTGGAATCTGAAT	184	1456
GAPDH	GCACGCCATCACTATCTTCC	CATCCACCGTCTTCTGTGTG	356	876

a Primers for AvBD4-13 and GAPDH are adopted from reference 14.

### Cell cytotoxicity of butyrate in HD11 cells

The cytotoxicity assay was performed as described previously [25], [32], [33]. Briefly, HD11 cells (1×10^5^) were seeded overnight in 96-well tissue culture plates. Butyrate was added in duplicate from 0 to 16 mM for 18 h, following by addition of 10% of alamarBlue (Invitrgoen) for another 6 h. The fluorescence was read at 545 nm excitation and 590 nm emission. Cell death (%) was calculated as [1−(*F*_butyrate_−*F*_background_)/(*F*_control_−*F*_background_)]×100, where *F*_butyrate_ is the fluorescence of cells exposed to different concentrations of butyrate, *F*_control_ is the fluorescence of cells only, and *F*_background_ is the background fluorescence of 10% alamarBlue in cell culture medium without cells.

### Antibacterial activity of monocytes treated with butyrate

Following overnight adherence of PBMCs to cell culture dishes, chicken monocytes were replenished with fresh antibiotic-free RPMI 1640 and incubated with 0, 0.5, 1, 2, and 4 mM of sodium butyrate for 24 h. Cells were then scraped, stored at −80°C overnight, lysed with 1% Triton X-100, and centrifuged at 12,000× *g* for 10 min at 4°C. Serial 2-fold dilutions were then prepared from the cell supernatants and incubated with 2×10^4^ CFU of *Salmonella enteritidis* (ATCC 13076) in 20% Trypticase Soy Broth containing 1 mM NaH_2_PO_4_ and 25 mM NaHCO_3_ for 9 h in a 96-well plate at 37°C as described [34]. Bacterial turbidity was measured at OD_590 nm_ using an ELISA plate reader. Different concentrations of sodium butyrate were also directly added to *S. enteritidis* in the same growth medium to measure turbidity after 9 h incubation.

### Phagocytosis assay of HD11 cells

Phagocytosis of *S. enteritidis* phage type 13a by HD11 cells was measured as described with slight modifications [35]. After seeding 6×10^6^ cells in complete RPMI 1640 overnight in 60-mm tissue culture plates, HD11 cells were stimulated with and without 0.5, 1 or 2 mM sodium butyrate for 24 h. Cells (2.5×10^6^) were then incubated with 2.5×10^7^ CFU of *S. enteritidis* phage type 13a in 1 ml RPMI 1640 containing 5% chicken serum for 30 min at 37°C. To kill extracellular bacteria, cells were washed twice with ice-cold HBSS, re-suspended with 1 ml RPMI 1640 containing 100 µg/ml gentamicin for 1 h at 37°C. Cells were then lysed by incubating with 1% Triton X-100 for 15 min, serially diluted, and spread on Brilliant Green agar plates (Becton Dickinson) containing 20 µg/ml of nalidixic acid and incubated overnight at 37°C for enumeration.

### Oxidative burst assay of HD11 cells

The assay of oxidative burst activity was performed as previously described with slight modifications [36]. Briefly, HD11 cells were seeded at 1×10^5^ cells in a 96-well plate in complete RPMI 1640 and cultured overnight. After addition of 0, 0.5, 1, and 2 mM of sodium butyrate for 24 h, cells were washed with HBSS to remove antibiotics, replenished with fresh RPMI 1640 free of Phenol Red and antibiotics, and rested for 30 min. Phorbol 12-myristate 13-acetate (PMA, Sigma) and 2′,7′-dichlorodihydrofluorescein diacetate (DCFDA, Sigma) were added to cells to final concentrations of 0.5 µg/ml and 10 µM, respectively. The fluorescence was monitored at 485 nm excitation and 528 nm emission using FLx800 Multi-Detection Microplate Reader (Bio-Tek Instruments) 1 h after incubation at 37°C. The results were normalized against protein concentrations, which were measured using the Bradford assay (Bio-Rad) as per manufacturer's instructions.

### Flow cytometric analysis of MHC class I and II surface markers

Following stimulation with 4 mM butyrate, 1 µg/ml LPS from *E. coli* O111:B4 (Sigma) or left untreated for 24 h, HD11 cells were scraped, washed, and adjusted to 1×10^6^/ml with the FACS buffer (0.1% BSA+0.02% sodium azide in phosphate buffered saline). Cells were preincubated in the FACS buffer containing 1% chicken serum and 1% of rat FCγ III/II receptor blocker (clone 2.4G2, eBioscience) for 15 min, followed by incubation with fluorescein isothiocyanate (FITC)-conjugated mouse anti-chicken MHC class I (clone F21-2, SouthernBiotech) and R-phycoerythrin (R-PE)-conjugated mouse anti-chicken MHC class II (clone 2G11, SouthernBiotech) monoclonal antibodies for another 30 min. Flow cytometry was performed on a FACSCalibur Flow Cytometer (Becton-Dickinson) and analyzed with BD CellQuest Pro-software.

### Butyrate feeding and *S. enteritidis* infection of chickens

Two chicken trials were conducted to test the in vivo effect of butyrate on HDP gene expression and disease resistance. In trial 1, a total of 20, five-day-old male Cornish Rock broiler chickens (Ideal Poultry, Cameron, TX) were equally divided and fed with a standard antibiotic-free ration mixed with or without 0.2% sodium butyrate for 48 h prior to intraesophageal infections with 0.5 ml of Lysogeny broth (LB) containing 1×10^6^ CFU of *S. enteritidis* phage type 13a [37]. After continuous feeding with butyrate-supplemented feed for another 4 days, the birds were euthanized and cecal contents were aseptically collected from each animal, serially diluted in PBS, and plated on Brilliant Green agar plates (Becton Dickinson) containing 20 µg/ml of nalidixic acid for bacterial enumeration. Trial 2 was conducted similarly with a total of 30, five-day-old male broilers fed with or without 0.1% or 0.2% sodium butyrate supplementation in the feed for two days, with 10 chickens per treatment. An intraesophageal infection with 1×10^6^ CFU of *S. enteritidis* phage type 13a was conducted 2 days later and butyrate supplementation was continued for another 4 days. Cecal contents were then collected from each chicken for bacterial counting. Unpaired Student's *t*-test was performed among groups, and p<0.05 was considered statistically significant.

## Results

### Butyrate induces HDP gene expression in chicken HD11 macrophage cells, primary monocytes, bone marrow cells, and jejunal and cecal explants

**Figure 1:**
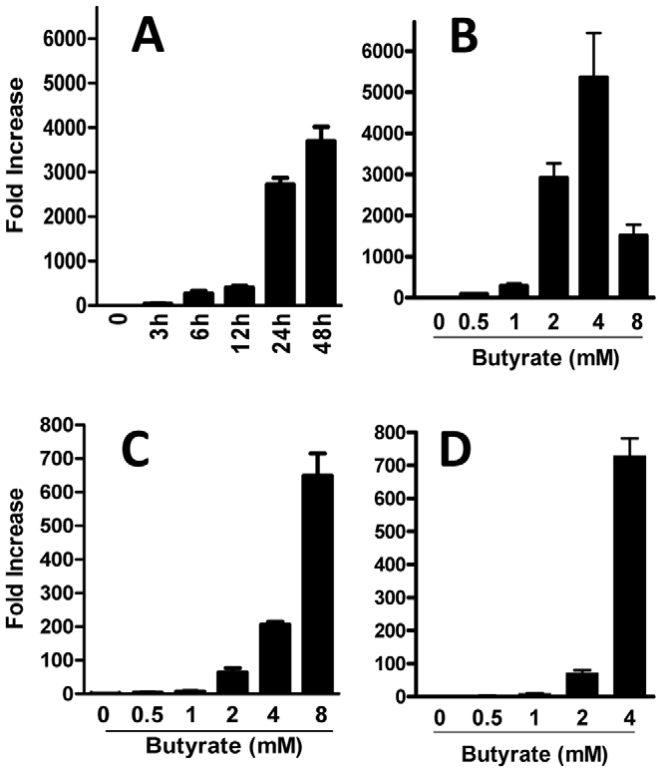
Butyrate-induced expression of the *AvBD9* gene in different chicken cell types. HD11 macrophage cells were incubated in duplicate with 4 mM sodium butyrate for indicated time points (A) or indicated concentrations of butyrate for 24 h (B). Chicken primary monocytes (C) or bone marrow cells (D) were exposed to different concentrations of butyrate in duplicate for 24 h prior to isolation of total RNA. The *AvBD9* gene expression was analyzed by real-time RT-PCR, and the relative fold increase over the control group was calculated using the comparative ΔΔCt method and the *GAPDH* gene for normalization. The bars represent means ± standard error of the data from 2–3 independent experiments.

To elucidate the effect of butyrate on HDP gene expression in the chicken, we first stimulated HD11 macrophage cells and primary chicken monocytes with different concentrations of sodium butyrate for various times, followed by real-time RT-PCR analysis of the expressions of the genes for all 14 AvBDs and 4 cathelicidins. The avian β-defensin 9 (*AvBD9*) gene was dramatically induced in HD11 cells in a time-dependent manner peaking at 24–48 h following stimulation with 4 mM butyrate (Fig. 1A). A dose-dependent induction was also evident in HD11 cells, with 4 mM butyrate giving nearly 5400-fold induction of *AvBD9* after treatment for 24 h (Fig. 1B). Similarly, the *AvBD9* gene expression was dose-dependently augmented in primary monocytes, resulting in a 200- and 650-fold increase following 24 h stimulation with 4 and 8 mM butyrate, respectively (Fig. 1C). A 700-fold augmentation of the *AvBD9* gene was also observed in chicken bone marrow cells treated with 4 mM butyrate for 24 h (Fig. 1D). It is noteworthy that the kinetics of butyrate-mediated HDP gene expression is similar in humans, where a peak response occurred in intestinal cell lines 1–2 days following treatment with 4 mM butyrate [29], [30]. However, it is not clear why the sensitivity of the two chicken cell types to butyrate differs. Butyrate at 4 mM gave an optimal induction of the *AvBD9* gene in HD11 and bone marrow cells, whereas a peak response occurred at 8 mM in primary monocytes, although no appreciable impact on the viability of the cells was observed in any cell type in response to up to 8 mM butyrate (data not shown).

**Figure 2:**
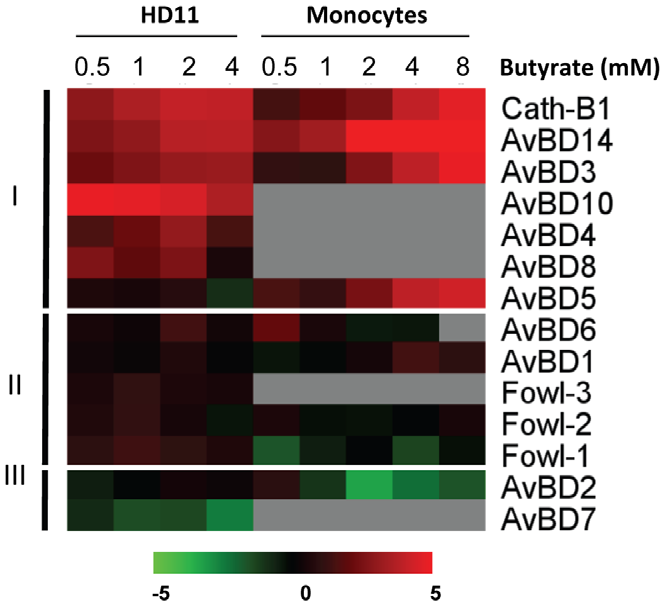
Induction of HDP gene expression in chicken HD11 macrophages and primary monocytes. Chicken HD11 macrophage cells and primary monocytes were incubated in duplicate with and without different concentrations of butyrate for 24 h, followed by RNA isolation and real-time RT-PCR analysis of all 14 chicken β-defensins (*AvBDs*) and 4 cathelicidins (*fowlicidins 1–3* and *cathelicidin B1*). The color elements represent average log_2_ ratios of fold change from 2–3 independent experiments. Red indicates up-regulation, whereas black means no induction and green down-regulation. Gray areas are an indication of no data due to extremely low expression levels of certain HDPs in primary monocytes. Three groups of chicken HDPs, namely generally induced (I), non-regulatable (II), and generally down-regulated (III), can be classified according to their mode of modulation by butyrate. *AvBD11*, *AvBD12*, and *AvBD13* could not be reliably detected in either cell type, and therefore, were not shown. The heat map was generating by using MultiExperiment Viewer [48].

Besides AvBD9, several other chicken HDP genes including *cathelicidin B1*, *AvBD3*, *AvBD4*, *AvBD8*, *AvBD10*, and *AvBD14*, also showed largely dose-dependent inductions in response to butyrate treatment in HD11 cells, albeit at a lesser magnitude than *AvBD9* (Fig. 2). A similar trend also occurred in chicken primary monocytes, where butyrate triggered dose-dependent up-regulation of *cathelicidin B1*, *AvBD3*, *AvBD5*, and *AvBD14* (Fig. 2). Notably, a subset of HDP genes including *AvBD1*, *AvBD6*, and *fowlicidins 1–3* were essentially not modulated by butyrate in either cell type (Fig. 2). Furthermore, *AvBD2* and *AvBD7* were even slightly down-regulated in primary monocytes and HD11 cells, respectively (Fig. 2), suggesting differential regulation of HDPs by butyrate.

**Figure 3:**
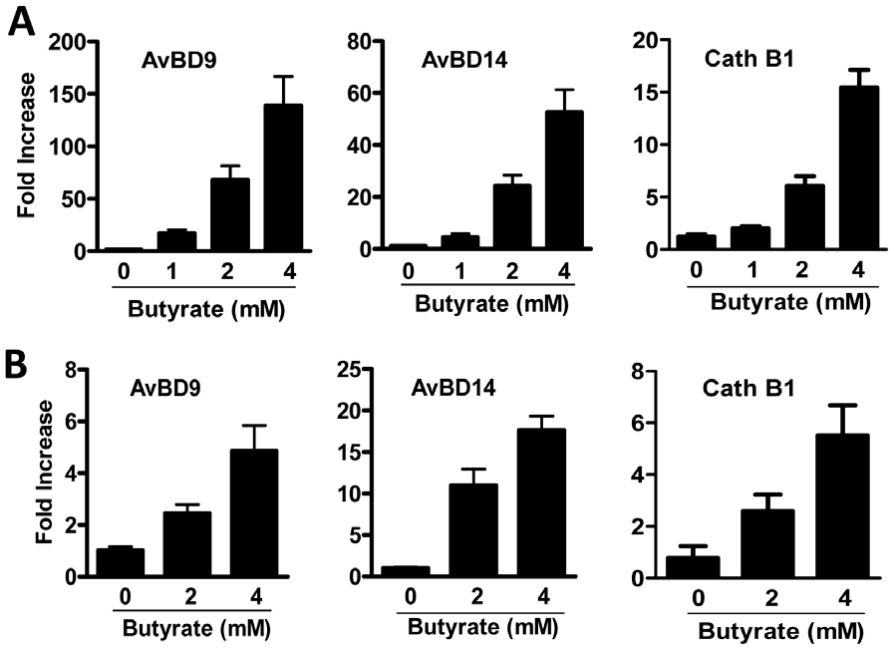
Up-regulation of three representative HDPs in chicken jejunal (A) and cecal explants (B) by butyrate. Chicken jejunum and cecal explants were obtained by culturing slices of 0.5 cm long segments, followed by incubation with indicated concentrations of butyrate in duplicate for 24 h. Real time RT-PCR was performed and the relative fold increase over the control group was calculated using the comparative ΔΔCt method and the *GAPDH* gene for normalization. The bars represent means ± standard error of the data from two independent experiments.

To further examine whether butyrate is capable of augmenting HDP gene expression in intestinal cells, chicken jejunal and cecal explants were prepared and stimulated with butyrate for 24 h. Three representative HDPs, namely *AvBD9*, *AvBD14*, and *cathelicidin B1*, were induced in a dose-dependent manner in both the jejunum (Fig. 3A) and cecum (Fig. 3B), although the magnitude of induction was generally less pronounced in the cecum than in the jejunum.

**Figure 4:**
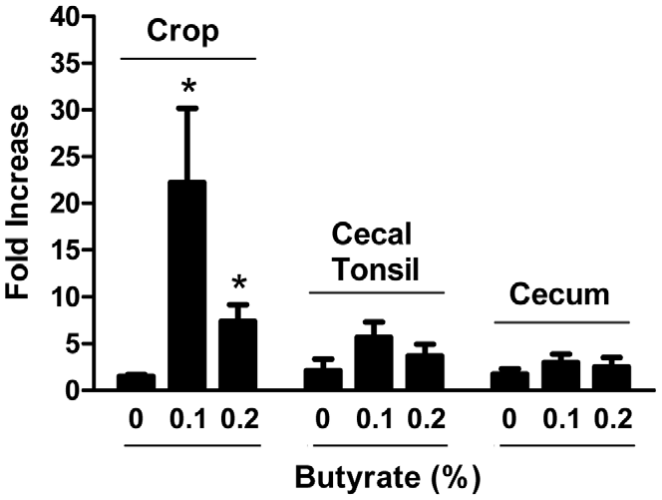
In vivo induction of the *AvBD9* gene expression in the intestinal tract of chickens by butyrate. Two-day-old male Cornish Rock broilers were fed with standard ration with or without supplementation of 0.1% and 0.2% butyrate for 2 days. The crop, cecal tonsil, and cecum were collected from each chicken and the *AvBD9* gene expression was evaluated by real-time PCR. Each bar represents means ± standard error of the data from 6 different chickens. * P<0.05 by unpaired Student's *t*-test.

To confirm the HDP-inducing activity of butyrate *in vivo*, we fed 2-day-old broiler chickens with and without 0.1% and 0.2% butyrate in standard ration for 2 days and harvested the crop, cecal tonsil, and cecum for real-time RT-PCR analysis of the *AvBD9* gene expression. As shown in Fig. 4, significantly induced *AvBD9* expression was observed in the crop, with 0.1% and 0.2% butyrate leading to 22- and 7.5-fold increase, respectively. A similar, but less dramatic trend also occurred in the cecal tonsil and cecum (Fig. 4). It is not known why a reduced response was seen with 0.2% butyrate supplementation compared to 0.1% butyrate. Perhaps higher concentrations of butyrate are more potent in inducing growth arrest and apoptosis [26], [27]. The finding that AvBD9 is induced more pronounced in the crop than in the lower digestive tract is perhaps related to tissue specificity. However, it is more likely because local concentrations of supplemented butyrate are much higher in the crop than in other parts of the intestinal tract, similar to earlier findings that the majority of butyrate is absorbed in the crop before reaching the lower digestive tract [38], [39]. Collectively, these results strongly suggest that butyrate is a potent inducer of the chicken HDP expression in multiple cell types both in vivo and in vitro, although cell- and tissue-specific induction patterns are also evident.

### Butyrate triggers no or minimum inflammatory response

**Figure 5:**
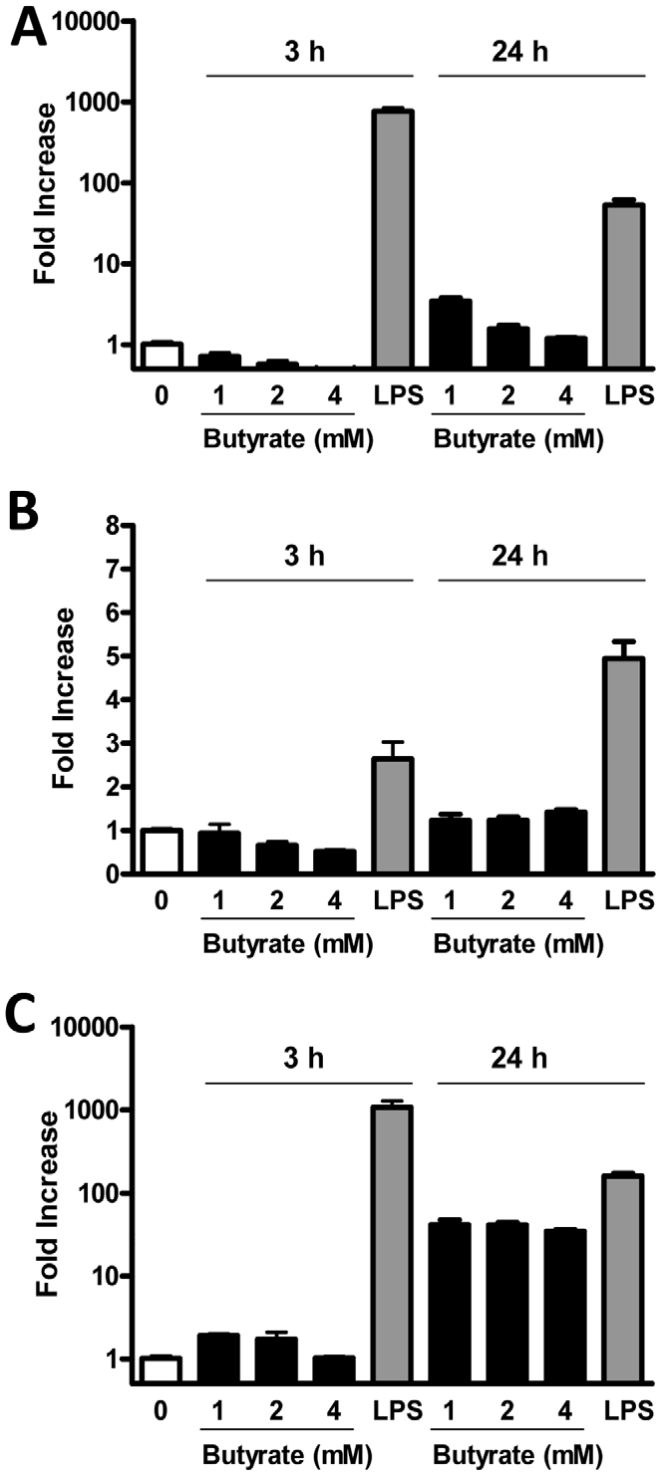
Minimum triggering of proinflammatory cytokine synthesis in HD11 cells by butyrate. Chicken HD11 macrophage cells were incubated with indicated concentrations of butyrate or 1 µg/ml LPS in duplicate for 3 and 24 h, followed by real-time PCR analysis of the gene expressions of *IL-1β* (A), *IL-12p40* (B), and *IL-8* (C). The bars represent means ± standard error of the data from two independent experiments. Essentially no induction of *IL-1* and *IL-12p40* was observed at both 3 and 24 h after butyrate stimulation, with moderate induction of *IL-8* occurring only following butyrate treatment for 24 h.

Butyrate generally exerts anti-inflammatory effects and has been used to treat inflammatory bowel diseases [26], [27]. To confirm butyrate-mediated specific augmentation of HDP gene expression without triggering a proinflammatory response, we treated HD11 cells with and without butyrate for 3 and 24 h and analyzed the expressions of three representative cytokines, namely IL-1β, IL-8, and IL-12p40. Butyrate had essentially no effect on either *IL-1β* (Fig. 5A) or *IL-12p40* expression (Fig. 5B) at both time points. No influence on *IL-8* expression was observed after 3 h stimulation with a moderate induction only after 24 h (Fig. 5C). In contrast, *IL-1β*, *IL-8*, and *IL-12p40* were induced markedly in response to 1 µg/ml LPS (Fig. 5). These results demonstrated that butyrate selectively induces HDPs with a minimum impact on proinflammatory cytokine expression, consistent with earlier transcriptional profiling results that butyrate is generally anti-inflammatory, suppressing expression of certain cytokines with no effect on the majority of them [40], [41].

### Butyrate augments the antibacterial activity of chicken monocytes through induction of HDPs

**Figure 6:**
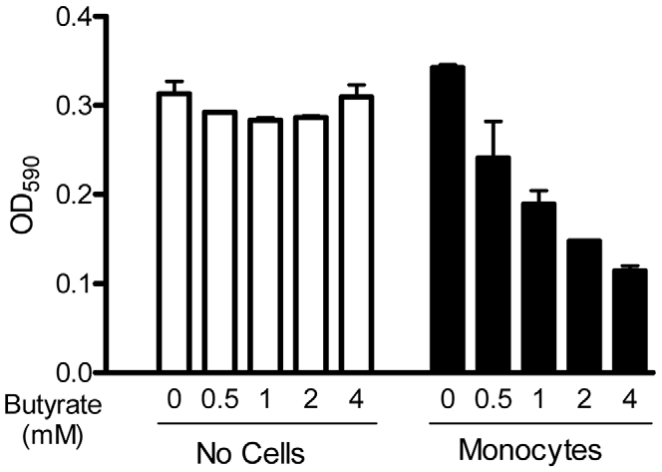
Augmentation of the antibacterial activity of monocytes following stimulation with butyrate. Chicken monocytes were treated with or without different concentrations of butyrate for 24 h. Cell lysates were then prepared and incubated with *S. enteritidis* (ATCC 13076) for 9 h at 37°C. Bacterial turbidity at OD_590 nm_ was measured as an indication of the bacterial density. *S. enteritidis* was also directly incubated with different concentrations of butyrate in cell culture medium alone without monocytes as controls (white bars). The bars represent means ± standard error of the data from two independent experiments.

To investigate the functional consequence of butyrate-induced HDP expression, we stimulated chicken primary monocytes with and without different concentrations of butyrate for 24 h, lysed cells, incubated cell lysates with *S. enteritidis*, and measured bacterial turbidity after 9 h. As shown in Fig. 6, a dose-dependent suppression of bacterial growth in butyrate-treated monocyte lysates was observed, with 4 mM butyrate giving greater than 3-fold reduction in turbidity. It is worth noting that incubation of bacteria with butyrate alone had not impact on bacterial growth at up to 4 mM (Fig. 6), implying that butyrate is incapable of killing bacteria directly at the HDP-inducing concentrations. Furthermore, given that butyrate in the cell culture medium was completely washed off prior to cell lysis and the antibacterial assay, an enhancement in the antibacterial activity of the cell lysates is unlikely due to the direct bacterial killing activity of butyrate.

**Figure 7:**
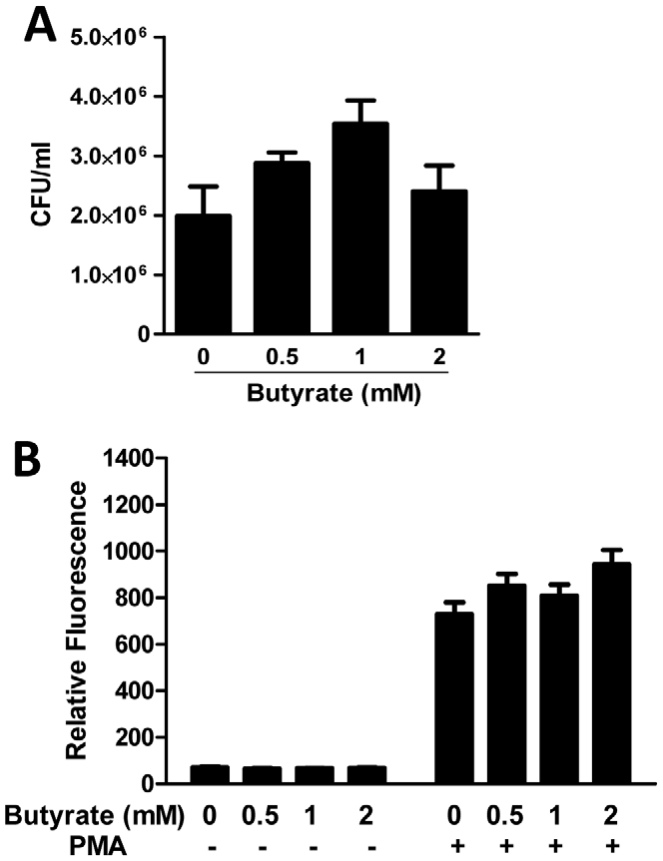
No impact of butyrate on phagocytic (A) or oxidative burst activities (B) of HD11 cells. In the phagocytosis assay, chicken HD11 macrophage cells were incubated with different concentrations of butyrate in duplicate for 24 h, followed by exposure to *S. enteritidis* phage type 13a for 30 min at 37°C in the presence of 5% chicken serum. Extracellular bacteria were then killed by gentamicin, and internalized bacteria were enumerated from lyzed HD11 cells by serial plating on Brilliant Green agar plates containing 20 µg/ml nalidixic acid overnight at 37°C. In the oxidative burst assay, HD11 cells were stimulated with indicated concentrations of butyrate for 24 h. The fluorescence was monitored at 485 nm excitation and 528 nm emission following 1 h incubation with 2′,7′-dichlorodihydrofluorescein diacetate (DCFA) in the presence or absence of phorbol 12-myristate 13-acetate (PMA). The results were normalized against protein concentrations of each sample. The bars represent means ± standard error of the data from two independent experiments.

To further rule out the possibility that butyrate-induced augmentation of the antibacterial activity was not attributed to a change in phagocytosis of chicken macrophages by butyrate, we first incubated HD11 cells with different concentrations of butyrate for 24 h and then measured the phagocytic capacity of the cells to *S. enteritidis*. In comparison with non-treated cells, essentially no difference in phagocytosis was observed with any concentration of butyrate (Fig. 7A). We further examined the influence of butyrate on the oxidative burst activity of chicken macrophages. As seen in Fig. 7B, PMA triggered a significant oxidative burst in HD11 cells; however, butyrate had a minimum impact on the cells treated with and without PMA.

**Figure 8:**
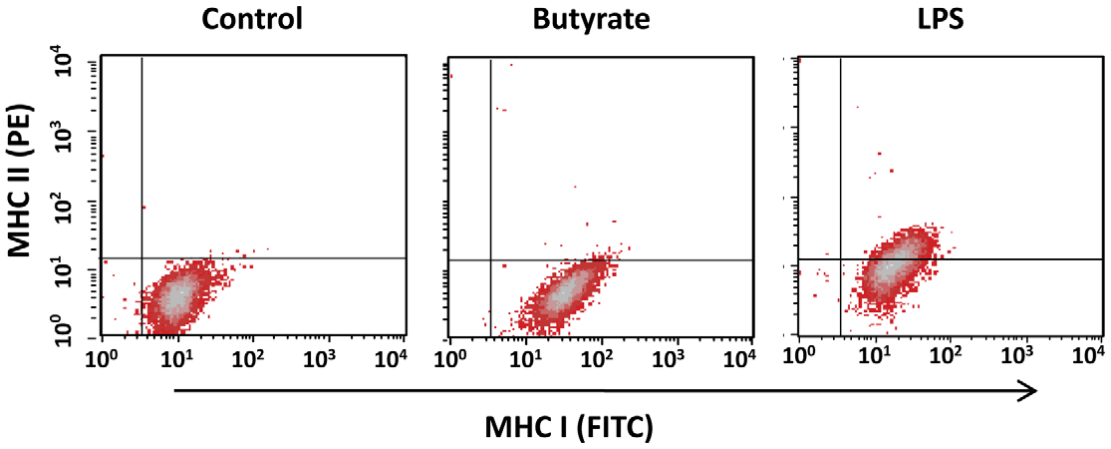
No influence on the activation status of HD11 cells by butyrate. HD11 cells were incubated with 4 mM butyrate, 1 µg/ml LPS or left untreated for 24 h, followed by flow cytometric analysis of surface expression of MHC class I and II using fluorescein isothiocyanate (FITC)-conjugated anti-chicken MHC class I and R-phycoerythrin (R-PE)-conjugated anti-chicken MHC class II monoclonal antibodies. The data shown are representative of two independent experiments.

To test whether butyrate is capable of activating chicken macrophages, we quantified a surface marker of cell activation, i.e., MHC class II, on HD11 cells by flow cytometry following stimulation with 2 mM butyrate for 24 h, using MHC class I as a house-keeping control. As expected, LPS stimulation induced surface expression of MHC class II in nearly 50% cells; however, essentially no change in MHC class II expression was observed in butyrate-treated HD11 cells (Fig. 8). These results collectively indicated that butyrate is incapable of modulating phagocytosis, oxidative burst or activation status of macrophage cells. Augmentation of the antibacterial activity in response to butyrate treatment, therefore, is likely due to specific induction of endogenous synthesis of HDPs.

### Oral supplementation of butyrate reduces *S. enteritidis* colonization in the cecum of infected chickens

**Figure 9:**
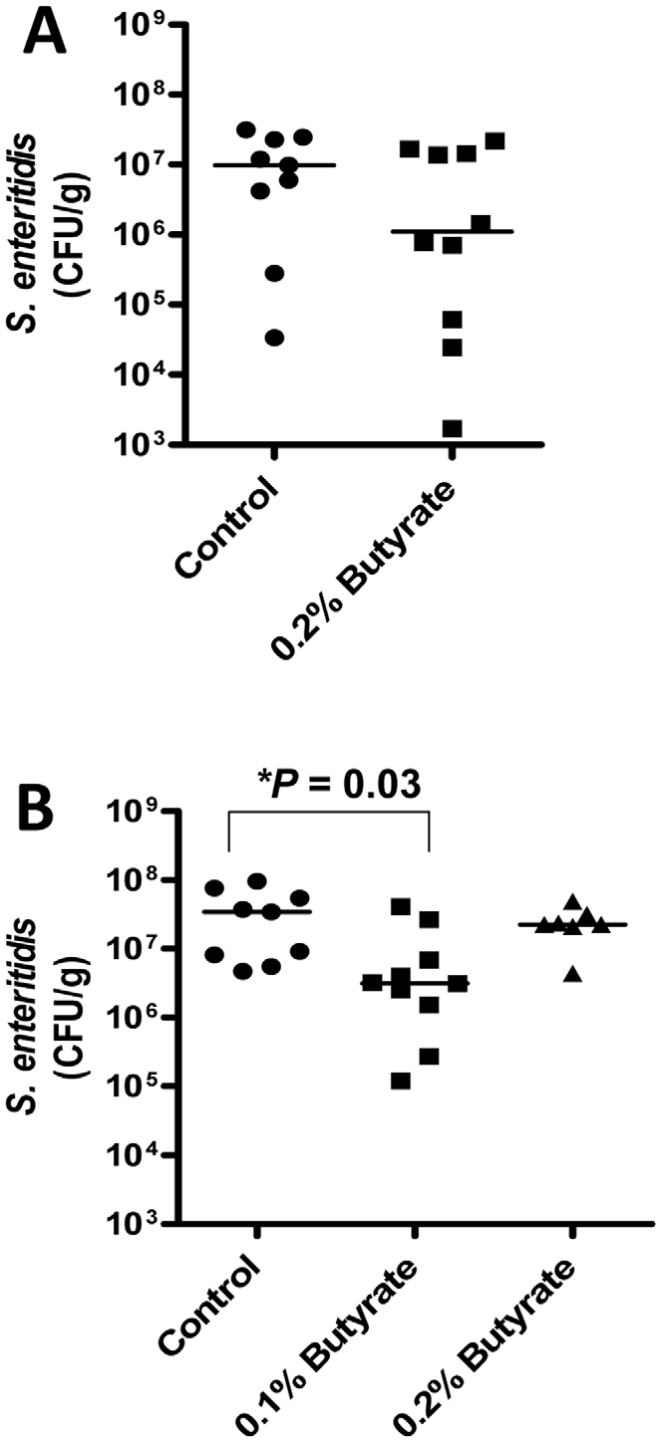
Reduction of the *S. enteritidis* titer in the cecal contents of chickens following oral supplementation of butyrate. In trial 1 (A), 5-day old male broilers were equally divided into two groups of 10 and fed with a standard antibiotic-free diet mixed with and without 0.2% sodium butyrate for 2 days. Birds were then inoculated with 1×10^6^ CFU of *S. enteritidis* phage type 13a and continued with butyrate feeding for another 4 days. The *S. enteritidis* titer in the cecal content was quantitated from each animal by serial plating on Brilliant Green agar plates containing 20 µg/ml nalidixic acid. Trial 2 (B) was similarly conducted with an additional group of 10 broilers fed with 0.1% butyrate. Each dot represents the bacterial titer from a bird and the solid line represents the median value of each treatment. Brackets indicate the statistical significance of differences (*P = 0.03, unpaired Student's *t*-test).

Because enhanced HDP gene expression and antibacterial activities were observed in cells in response to butyrate treatment, we evaluated whether supplementation of feed with butyrate can reduce the survival of pathogenic bacteria in the intestinal tract of 5-day-old broilers in two separate trials. Chickens were fed with and without 0.1% and/or 2% butyrate for 2 days prior to intraesophageal inoculation of *S. enteritidis* phage type 13a for another 4 days. The cecal contents, where *S. enteritidis* most heavily colonizes, were aseptically harvested and subjected to serial plating on Brilliant Green agar plates containing 20 µg/ml of nalidixic acid for specific enumeration of *S. enteritidis* 13a. In trial 1, oral supplementation of 0.2% butyrate resulted in 1-log reduction in the median counts of inoculated bacteria in the cecal content, relative to the control group (Fig. 9A). In trial 2, 0.1% butyrate significantly reduced bacterial load (*P* = 0.03) in the cecal content of the chickens, whereas 0.2% butyrate led to a less reduction of bacterial counts (Fig. 9B). This is perhaps not surprising, given the earlier findings that, as compared to 0.1% butyrate, 0.2% butyrate supplementation caused less induction of the HDP genes in the intestinal tract (Fig. 4).

## Discussion

As a major species of short-chain fatty acids produced from fermentation of undigested dietary fiber by intestinal microflora, butyrate exerts a plethora of effects on intestinal health and disease [26], [27], [39]. In addition to being a primary energy source for colonocytes in mammals, butyrate has been found to play an important role in the digestive tract by stimulating mucin synthesis and intestinal motility, cell proliferation and differentiation, while suppressing inflammatory diseases [26], [27], [39]. In the present study, we have revealed a novel role for butyrate in host defense and extended earlier findings that butyrate-induced synthesis of HDPs not only occurs in humans and rabbits [28], [29], [30], but is also conserved in chickens. We have presented both in vitro and in vivo evidence showing that butyrate strongly induces the expressions of multiple HDPs in different cell and tissue types including HD11 macrophages, primary monocytes, bone marrow cells, jejunum and cecal explants as well as in crop, cecum, and cecal tonsils of chickens. The results clearly suggest that transcriptional regulatory mechanisms of many HDPs are phylogenetically conserved across mammals and aves.

It is important to note that only a subset of chicken HDPs are regulated by butyrate (Fig. 2), implying that HDPs are differentially regulated even within the same family. Consistently, only LL-37 and human β-defensin-2 were reported to be regulated by butyrate in humans [29], [30], [42]. For those chicken HDP genes that are modulated by butyrate, we observed a clear cell-specific regulation pattern as evidenced by marked differences in the magnitude of induction among different cell types. For example, treatment with 4 mM butyrate for 24 h induced the *AvBD9* gene approximately 3,000- to 5,000-fold in HD11 macrophage cells, but only 200-fold in primary monocytes, 700-fold in bone marrow cells, 140-fold in jejunal explants, and 5-fold in cecal explants (Figs. 1 and 3). Several other HDPs, e.g., *AvBD14* and *cathelicidins B1* were also regulated differently among individual cell types (Fig. 3 and data not shown).

Although we could not detect the synthesis of chicken HDPs at the protein level in response to butyrate treatment due to a lack of specific antibodies, we observed an increased HDP gene synthesis leading to an enhanced antibacterial activity in monocytes in vitro and augmented intestinal bacterial clearance in vivo following butyrate treatment. A nearly 10-fold reduction in the bacterial titer was achieved in the cecal contents of the chickens fed 0.1% or 0.2% butyrate (Fig. 9). Given the rapid rate of absorption and metabolism, the majority of supplemented butyrate is known to be taken up by the upper digestive tract, with very small quantities reaching the lower intestinal tract or general circulation [38], [39]. A more pronounced reduction in the cecal bacterial titer may be achieved if supplemented butyrate can be protected when passing through the upper digestive tract or if more butyrate can be produced in the cecum by manipulating the conditions of local bacterial fermentation [38], [39].

It is noteworthy that 0.1% butyrate gave a better bacterial reduction than 0.2% butyrate in our feeding trial (Fig. 9B), in agreement with the finding that 0.1% butyrate supplementation led to a higher level of the *AvBD9* gene transcription in the crop, cecum, and cecal tonsil of chickens than 0.2% butyrate (Fig. 4). Consistently, 8 mM butyrate failed to stimulate the synthesis of a higher amount of the *AvBD9* transcripts in HD11 cells than 4 mM butyrate (Fig. 1B). In fact, higher concentrations of butyrate often lead to cytotoxicity, growth arrest, and apoptosis [26], [27], [39]. The optimal dose of butyrate for in vivo applications, therefore, needs to be investigated carefully for each animal species.

It was reported earlier that oral supplementation of 0.63 mg/kg or 0.92 mg/kg of butyrate reduces colonization and shedding of *S. enteritidis* in the cecum of chickens [43], [44]. However, the mechanism by which butyrate suppresses bacterial growth remain elusive, although it was proposed to be a result of the direct antibacterial activity of butyrate [45], [46] or a decrease in the invasiveness of *Salmonella* through intestinal epithelial cells following exposure to butyrate [35], [46]. However, because especially high concentrations of butyrate (25, 50, and 100 mM) were needed to kill bacteria or negatively impact on bacterial invasiveness [35], [45], [46], it is uncertain whether these proposed mechanisms may occur in vivo, given that most butyrate is absorbed in the upper digestive tract if supplemented orally [38], [39] and that cecal concentrations of butyrate are only <6 mM in 18-day-old healthy broiler chickens and <1 mM in 4-day-old chickens [46]. More importantly, an increased invasion to intestinal epithelial cells was observed in the same study when *S. enteritidis* was pre-incubated with a mixture of short-chain fatty acids mimicking the in vivo cecal concentrations [46]. Here, we uncovered a novel mechanism that we believe accounts primarily for butyrate-mediated suppression of intestinal bacterial colonization. We found that at physiological concentrations butyrate fails to inhibit bacteria directly, but increase the antibacterial activity of host innate immune cells by inducing the synthesis of an array of HDPs with a minimum impact on the phagocytic and oxidative killing capacity as well as activation status of host cells. Therefore, it is the production of HDPs that is mainly responsible for a reduction of bacterial colonization in the intestinal tract of chickens following oral supplementation of butyrate.

Our in vitro and in vivo studies have firmly established that butyrate has a strong capacity to induce HDP synthesis and that supplementation of butyrate can augment disease resistance and reduce bacterial colonization in chickens. Therefore, the strategies for efficient delivery of butyrate to the lower intestinal tract will have important implications in animal health and food safety. Indeed, the microencapsulated form of butyrate proves to be more efficient in suppressing bacterial growth in the ceca of chickens than the free unprotected form [43], [44]. Alternatively, identification and application of less labile forms of butyrate analogs in the feed may also prove to be more desirable. In fact, several butyrate analogs have been shown to be capable of inducing HDP gene expression in humans [47] and such analogs await further testing for their antibacterial efficacy in other animal species such as chickens. Besides direct administration of butyrate and its analogs, the dietary approaches that promote the proliferation of butyrate-producing bacteria and stimulate the fermentation of butyrate through the use of prebiotics may also have good prospect to augment HDP synthesis and host defense.

In summary, we have revealed that butyrate-induced synthesis of endogenous HDPs is a phylogenetically conserved mechanism of innate host defense shared by both mammals and chickens. Moreover, we propose that butyrate-induced HDP synthesis represents a newly discovered mechanism that mainly accounts for the suppression of bacterial colonization and shedding in farm animals by butyrate. Coupled with anti-inflammatory effects and other beneficial properties, butyrate, butyrate analogs, and perhaps other short-chain fatty acids may have potential for further development as antibiotic-alternative food or feed additives to boost innate immunity and disease resistance of humans and animals without provoking a harmful proinflammatory response.
